# Gastrointestinal parasites of wild carnivores from conservation institutions in the Cerrado of Goiás, Brazil

**DOI:** 10.1590/S1984-29612023028

**Published:** 2023-05-22

**Authors:** Renan Mendes Pires Moreira, Caroline Genestreti Aires, Ana Vitória Alves-Sobrinho, Iago de Sá Moraes, Cecília Nunes Moreira, Andréia Vitor Couto do Amaral, Klaus Casaro Saturnino, Ísis Assis Braga, Richard de Campos Pacheco, Dirceu Guilherme de Souza Ramos

**Affiliations:** 1 Programa de Pós-graduação em Biociência Animal, Unidade Acadêmica de Ciências Agrárias, Universidade Federal de Jataí – UFJ, Jataí, GO, Brasil; 2 Laboratório de Parasitologia e Análises Clínicas Veterinária, Unidade Acadêmica de Ciências Agrárias, Universidade Federal de Jataí – UFJ, Jataí, GO, Brasil; 3 Laboratório de Anatomia Patológica Veterinária, Unidade Acadêmica de Ciências Agrárias, Universidade Federal de Jataí – UFJ, Jataí, GO, Brasil; 4 Unidade Básica das Biociências, Centro Universitário de Mineiros, Mineiros, GO, Brasil; 5 Programa de Pós-graduação em Ciências Veterinárias, Faculdade de Medicina Veterinária, Universidade Federal de Mato Grosso – UFMT, Cuiabá, MT, Brasil

**Keywords:** Ancylostomatidae, Carnivora, conservationism, coproparasitological test, zoonosis, Ancylostomatidae, Carnivora, conservacionismo, teste coproparasitológico, zoonose

## Abstract

Increased interaction between wild and urban environments owing to human population growth, increased anthropization of biomes, and habitat loss for wild animals increases the spread of infectious and parasitic agents. The present study reports on the occurrence of gastrointestinal parasites in carnivorous mammals at two conservation institutions in the state of Goiás, Brazil. Fecal samples from 39 adult carnivores were collected after spontaneous defecation and analyzed by flotation and sedimentation. The structure and management data of each institution were recorded. Parasitism prevalence, binomial confidence intervals (CI) at 95%, variables associated with the presence of contact animals, size of the enclosure and type of food were recorded. The overall prevalence of gastrointestinal parasites in the samples analyzed was 71.8% (CI 55.1–83.0; 28/39). Ancylostomatidae, *Toxocara* spp., *Toxascaris leonina*, *Strongyloides* spp., *Calodium hepaticum*, and Trematoda eggs, and *Cystoisospora* spp. oocysts were detected. Environmental conditions were not correlated with parasitism prevalence; however, the parasites found could be managed, considering their biology, such as controlling synanthropic and domestic animals in captivity, feeding with healthy feed.

## Resumo

A crescente urbanização e a perda de hábitat para animais selvagens tem levado a uma maior interação entre ambientes selvagens e humanos, o que pode favorecer a disseminação de agentes infecciosos e parasitários. O presente estudo relata a ocorrência de parasitas gastrointestinais em mamíferos carnívoros em duas instituições de conservação no estado de Goiás, Brasil. Amostras fecais de 39 carnívoros adultos foram coletadas após a defecação espontânea e analisadas por flotação e sedimentação. Foram registrados os dados de estrutura e gestão de cada instituição. Prevalência de parasitismo, intervalo de confiança binomial (IC) a 95% e variáveis associadas a presença de animais de contato, tamanho do recinto e tipo de alimento foram registrados. A prevalência geral de parasitas gastrointestinais nas amostras analisadas foi de 71,8% (IC 55,1–83,0; 28/39). Foram detectados ovos de Ancylostomatidae, *Toxocara* spp., *Toxascaris leonina*, *Strongyloides* spp., *Calodium hepaticum* e de Trematoda e oocistos de *Cystoisospora* spp.. As condições ambientais não apresentaram correlação com a prevalência de parasitismo; entretanto, os parasitos encontrados podem ser manejados, considerando-se sua biologia, como controlar animais sinantrópicos e domésticos em cativeiro, e uso de alimentos livres de contaminantes.

Medium-sized and large mammals perform various ecological functions, such as regulating the population of other animals in the food chain, dispersing seeds, and acting as ecosystem engineers. Among these, species of the order Carnivora that are at the top of the food chain can be indicators of good environmental quality as they regulate the functioning of ecosystems. Changes in the diversity and population of species of the order Carnivora can damage the trophic chain through competition and predation by other species (Oliveira et al., 2001).

The expansion of urban areas increases anthropization of biomes, increase in deforestation, the growth of urban areas, and animal production decrease the habitat of several species and promotes the occurrence of different diseases, particularly parasitic diseases (Sprenger et al., 2018). Several groups of pathogens with zoonotic potential affect wild carnivores (viruses, bacteria, protozoa, arthropods and helminths), and according to Otranto & Deplazes (2019), the risk of transmission of these pathogens to humans, especially helminths such as *Ancylostoma*, *Baylisascaris*, *Capillaria*, *Uncinaria*, *Strongyloides*, *Toxocara* and *Trichinella* that occur in wild carnivores and can be transmitted to humans via food, water or soil, has been neglected.

The Cerrado savanna is one of the richest biodiversity biomes worldwide and the second largest biome in Brazil. However, fragmentation owing to mechanized agriculture and the establishment of paddocks for livestock is affecting the Cerrado biome (Calaça et al., 2010). According to the Chico Mendes Institute of Biodiversity Conservation (Instituto Chico Mendes de Conservação da Biodiversidade, 2018), habitat loss is the major threat to Brazilian wildlife, followed by poaching.

The objective of the present study was to determine the gastrointestinal parasites in carnivorous mammals, from two conservation institutions in the state of Goiás, Brazil, through coproparasitological examinations.

Fecal samples of captive carnivore mammals were collected from the Goiânia Zoological Park (GZP), and from *Instituto Onça Pintada* (IOP), Goiânia and Mineiros respectively, Goiás, Brazil. The mean annual temperature varies between and 22.3-26.3°C and 19.3-24.7°C, whilst mean humidity of 40.8-72.6% and 49.7-78.4% are recorded for Goiania and Mineiros, respectively (Brasil, 2020).

The samples were collected directly from animal’s enclosure without handling the animals, along with enclosure maintenance and animal care procedures. Samples were collected in the morning immediately after defecation. During collection days, maintenance of the captivity was carried out when defecation was verified, to guarantee the non-development of larvae in the eggs; at each collection, we observed defecation and performed the collection, ensuring the sample from each animal with a minimum of handling. Thirty-nine adult carnivores of different species were sampled, as shown in Table 1, corresponding to 100% of animals available at the two institutions for the present study. The samples were placed in fecal collection pots, labeled, and refrigerated at 4 °C. Furthermore, data on the structure of the enclosure and management strategies adopted by each institution were recorded.

**Table 1:** Carnivores sampled for coproparasitological examination to identify gastrointestinal parasites from two conservation institutions in Goiás, Brazil.

**Species**	**IOP** *	**GZP** **	**Total**
	**(n=21)**	**(n=18)**	**(n=39)**
Felidae			
*Panthera tigris*	0	4	4
*Panthera leo*	0	2	2
*Panthera onca*	16	5	21
*Puma concolor*	1	1	2
*Leopardus pardalis*	0	4	4
*Herpailurus yagouaroundi*	0	1	1
Canidae			
*Chrysocyon brachyurus*	3	0	3
*Cerdocyon thous*	0	1	1
Procyonidae________________			
*Potos flavus*	1	0	1

* Instituto Onça Pintada;

** Goiânia Zoological Park.

Fecal samples were analyzed using the coproparasitological techniques of flotation and spontaneous sedimentation adapted by Hoffmann (1987), and analyzed under an optical microscope used for the detection of gastrointestinal parasites. Eggs and oocysts were measured with the aid of Moticam 3.0 coupled to Nikon E200 microscope 40x and 100x magnification, and identified according to Moravec (1982) and Foreyt (2013). The prevalence of gastrointestinal parasites was recorded considering location, host species, and parasite species, and the confidence interval (CI) for the total percentage of parasitized animals was calculated. The following variables associated with the presence of parasites were analyzed using the chi-square test: presence or absence of contact animals in the enclosures, enclosure size, and type of food. The difference between the number of positives based on flotation and sedimentation techniques was also compared.

The overall prevalence of gastrointestinal parasites in the samples analyzed was 71.8% (CI 55.1–83.0; 28/39). The prevalence of gastrointestinal parasites at the GZP was 77.8% (CI 55.4–93.3; 14/18), and in the IOP was 66.7% (CI 43.0–85.4; 14/21). Ancylostomatidae, *Toxocara* spp., *Toxascaris leonina*, *Strongyloides* spp., *Calodium hepaticum* (syn. *Capillaria hepatica*), and Trematoda eggs and *Cystoisospora* spp. oocysts were observed from the samples.

A higher environmental diversity was observed at the IOP than that at the GZP, with few sparsely vegetated areas, and many densely vegetated areas and lakes, and some felids practice exercises outside their territory. Seven animals lived in isolation, others had at least one contact, and maned wolves (*Chrysocyon brachyurus*) shared a habitat with tapirs (*Tapirus terrestris*). The other enclosures were shared among congener species. Animal fur and bones were observed in most animal enclosures. The diet consisted of white-lipped peccary (*Tayassu pecari*) meat raised at the same institution, slaughterhouse donations, and carcasses collected from wild animals run over by traffic on highways. The diets of canids and raccoons comprised meat and fruits.

In the GZP, the enclosures were similar. The habitat for large cats was vast, with trunks, platforms, lakes, and streams, with little or no vegetation. For smaller cats, the enclosure was small, with few vegetation, burrows, and small ponds. The environment, clothes, and shoes of the caregivers were sanitized daily. The animal diet consisted of red meat and chicken.

In both institutions, the presence of synanthropic animals in the enclosures (pigeons, rats and lizards) was observed. The presence of domestic animals near the enclosures, such as dogs and cats, was also observed in both institutions. None of the institutions described to us the origin of each animal. The two institutions have veterinary assistance, but the IOP did not carry out periodic examinations or treatments, only under suspicion of parasitic disease, while the GZP carried out coproparasitological examinations every six months and treatment in parasitized animals.

**Figure 1:**
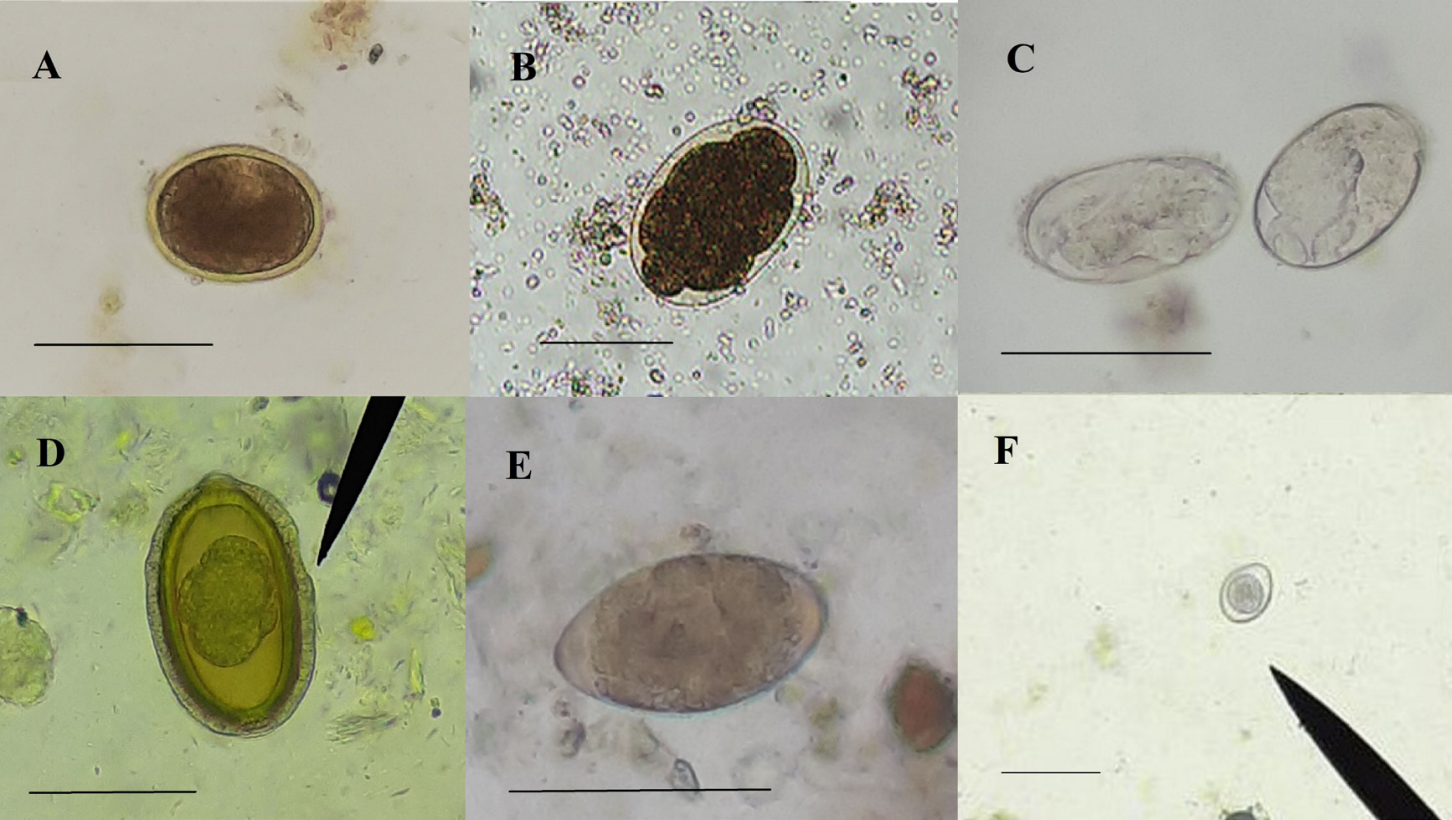
Eggs and oocysts of gastrointestinal parasites in stool samples from carnivorous mammals from the *Instituto Onça Pintada*, Brazil and Zoological Park of Goiânia, Goiás, Brazil. (A) *Toxocara* spp. egg (bar. 90µm), (B) Ancylostomatidae egg (bar. 35µm), (C) *Strongyloides* spp. egg (bar. 80µm), (D) *Calodium hepaticum* egg (bar. 30µm), (E) Trematoda egg (bar. 55µm) and (F) *Cystoisospora* spp. oocyst (bar. 50µm).

None of the variables analyzed was correlated with parasitosis (*p* > 0.05). No significant differences between the flotation and sedimentation techniques were noted. The results of the present study are shown in Tables 2 and 3. The helminth eggs and protozoan oocysts identified in the present study are shown in Figure 1.

**Table 2:** Occurrence of gastrointestinal parasites at the Instituto Onça Pintada and the Goiânia Zoological Park, Goiás separately by institution.

**Species**	**Conservation Institute in Mineiros**			**Goiânia Zoological Park**	
	**N**	**Parasite (n+)**		**N**	**Parasite (n+)**
Felidae					
*Panthera tigris*	-	-		4	*Toxocara* spp (1)
					*Cystoisospora* spp (2)
*Panthera leo*	-	-		2	-
*Panthera onca*	16	Ancylostomatidae (1)		5	Ancylostomatidae (1)
		*Strongyloides* spp. (1)			*Toxocara* spp. (2)
		Trematoda (10)			*Cystoisospora* spp. (4)
*Puma concolor*	1	*Toxascaris leonina* (1)		1	-
		Trematoda (1)			
*Leopardus pardalis*	-	-		4	Ancylostomatidae (1)
					*Toxocara* spp. (2)
					Trematoda (1)
					*Cystosospora* spp. (1)

*Herpailurus yagouaroundi*	-	-		1	*Toxocara* spp. (1)
					
Canidae					
*Chrysocyon brachyurus*	3	*Calodium hepaticum* (1)			
		Trematoda (1)			
*Cerdocyon thous*	-	-		1	*Cystoisospora* spp. (1)
					
Procyonidae					
*Potos flavus*	1	-		-	-

**Table 3:** Occurrence of gastrointestinal parasites at the Instituto Onça Pintada and the Goiânia Zoological Park, Goiás separately by parasite species.

**Species**	**Occurrence % (N_positive_/N_total_)**						
	Ancylostomatidae	***Toxocara* spp.**	***Toxascaris leonina***	***Strongyloides* spp.**	***Calodium hepaticum***	**Trematoda**	***Cystoisospora* spp.**
							
Felidae							
*Panthera tigris*	-	25.0 (1/4)	-	-	-	-	50.0 (2/4)
*Panthera leo*	-	-	-	-	-	-	-
*Panthera onca*	9.5 (2/21)	9.5 (2/21)	-	4.8 (1/21)	-	47.6 (10/21)	19.0 (4/21)
*Puma concolor*	-	-	50.0 (1/2)	-	-	50.0 (1/2)	-
*Leopardus pardalis*	25.0 (1/4)	50.0 (2/4)	-	-	-	25.0 (1/4)	25.0 (1/4)
*Herpailurus yagouaroundi*	-	100.0 (1/1)	-	-	-	-	-
							
Canidae							
*Chrysocyon brachyurus*	-	-	-	-	33.3 (1/3)	33.3 (1/3)	-
*Cerdocyon thous*	-	-	-	-	-	-	100.0 (1/1)
							
Procyonidae							
*Potos flavus*	-	-	-	-	-	-	-

Hookworms are geohelminths with a direct life cycle and can cause hemorrhagic gastroenteritis and in Brazil, the genus *Ancylostoma* is the most prevalent in domestic dogs and felines, leading to the contamination of many public places and is a health risk (Santos et al., 2020). Furthermore, the genus *Ancylostoma* has been reported in several wild animals (Aranda et al., 2013; Ramadevi & Venu, 2020). The occurrence of *Ancylostoma* in *Panthera onca* and *Leopardus pardalis* observed in the present study demonstrates the susceptibility of wild animals to parasitic species affecting domestic animals. Animals in captivity are infected owing to contamination of the environment by domestic animals living around the wild animal enclosures (Lo et al., 2021). Another recurrent hookworm species in wild carnivores is *Uncinaria stenocephala*, with several reports, especially in Europe, with higher prevalences than *Ancylostoma* species in some countries, and despite lesions similar to *Ancylostoma* in animals, its zoonotic potential is unclear (Otranto & Deplazes, 2019).

*Toxocara* spp. are zoonotic and cosmopolitan nematodes prevalent in young animals (Dantas-Torres, 2020). Reports of *P. onca*, *Panthera tigris*, *L. pardalis,* and *Herpailurus yagouaroundi* infection by *Toxocara* spp. are common and have been described in captive animals in other studies (Uribe et al., 2021). Infection occurs through environmental contamination through egg dispersal by infected animals. However, small rodents are paratenic hosts (Santos et al., 2009) and carry the parasites into the animal enclosures; thus, the occurrence of helminths in captive carnivores.

*Toxascaris leonina* are morphologically and biologically similar to *Toxocara* spp., and occurs less frequently in young animals, as there is no transmammary or transplacental transmission. Transmission by paratenic hosts is more common, as the *T. leonina* host range is bigger than that of other parasites, ranging from rodents to birds (Okulewicz et al., 2012). Therefore, adult animals are more easily infected since they hunt more successfully. *Toxascaris leonina* parasitizing on *Puma concolor* at the Conservation Institute of Mineiros, Brazil, is correlated with the consumption of carcasses of run-over animals recorded at the institution; however, infection through the contaminated environment is possible since some animals were exercised in the area surrounding the animal enclosures.

*Strongyloides* is a genus of parasitic nematodes with more than 50 species parasitizing the digestive system of vertebrates (amphibians, reptiles, and birds) and mammals, including humans (Viney & Lok, 2015). Most *Strongyloides* spp. are asymptomatic and self-limiting. However, low pathogenicity and high infection can cause diarrhea, fecal mucus, skin lesions, bronchopneumonia, intestinal nodules, and severe enteritis (Thamsborg et al., 2017). In the present study, *Strongyloides* spp. parasitism was observed in one *P. onca,* already reported previously (Aranda et al., 2013). Oliveira et al. (2001) listed the main felid parasites and revealed *Strongyloides* spp. parasitism in South American zoos, demonstrating the high adaptability of the *Strongyloides* genus to different regions, diverse environments, and various hosts.

*Calodium hepaticum* are widely distributed zoonotic nematodes parasitizing the liver of rodents, lagomorphs, mustelids, canids, felids, and primates, including humans (Misra et al., 2019). Adult *C. hepaticum* worms reproduce in the liver parenchyma cells. Females oviposit encapsulated eggs; therefore, are not released directly into the environment (Li et al., 2010; Misra et al., 2019). Therefore, *C. hepaticum* infection occurs after liver ingestion, predation, cannibalism, or carcass consumption or eggs released into the soil during host decomposition (Li et al., 2010). In the present study, *C. hepaticum* eggs were found in maned wolf (*C. brachyurus*) feces. Reports of *C. hepaticum* parasitism in maned wolves are limited, with one report by Massara et al. (2015) in a free-ranging wolf in the southwestern Brazil peri-urban region owing to a possible transmission between domestic dogs and wolves. Furthermore, *C. hepaticum* eggs in maned wolf feces do not always indicate parasitism. Owing to the supply of contaminated carcasses, the eggs present in the liver of these carcasses passed through the gastrointestinal tract of animals, and the infection may or may not have occurred. Wild animal carcasses are offered to the IOP where the animal lived presenting another possible transmission route.

Trematoda is a group of more than 24,000 species (Bolek et al., 2019), and their eggs are found in felids and other mammalian species (Basu & Charles, 2014). Trematoda infection of carnivores in the present study was correlated with the consumption of carcasses, small rodents, or synanthropic reptiles that may be present in the enclosures and function as intermediate or paratenic hosts of this group of helminths. Morphological differentiation of eggs of this group is difficult because of the morphological similarities between the eggs of the species.

In the present study, *Cystoisospora* spp. parasitizing *P. tigris*, *P. onca*, *L. pardalis*, and *C. thous* was observed. Several studies have reported *Cystoisospora* spp presence in wild felids. Lima et al. (2020) revealed that the presence of *Cystoisospora* spp. in *P. concolor*, *H. yagouaroundi*, and *Leopardus guttulus* correlate with the presence of feral cats, which are potential sources of infection for wild animals. Thus, managing captive feral cats in conservation institutions may control diseases in wild carnivores.

Helminth eggs and protozoan oocysts infecting domestic animals and humans were found in wild carnivores at the two conservation institutions sampled. Parasite management strategies for captive animals are recommended. Additionally, identifying possible synanthropic and domestic animals with access to captivity enclosures and their surroundings that serve as intermediate and paratenic hosts is recommended. Strategies to prevent parasitosis, such as providing adequate food and periodic diagnosis of parasites, are essential because captive animals are more susceptible to parasites than free-living animals.
